# Association between immunologic markers and cirrhosis in individuals with chronic hepatitis B

**DOI:** 10.1038/s41598-021-00455-8

**Published:** 2021-11-15

**Authors:** Ilona Argirion, Ruth M. Pfeiffer, Tram Kim Lam, Thomas R. O’Brien, Kelly Yu, Katherine A. McGlynn, Jessica L. Petrick, Ligia Pinto, Chien-Jen Chen, Mei-Hsuan Lee, Allan Hildesheim, Hwai-I Yang, Jill Koshiol

**Affiliations:** 1 Division of Cancer Epidemiology and Genetics, National Cancer Institute, Rockville, MD USA; 2 Division of Cancer Control and Population Sciences, National Cancer Institute, Rockville, MD USA; 3 HPV Immunology Laboratory, Frederick National Laboratory for Cancer Research, Leidos, Biomedical Research, Inc, Frederick, MD USA; 4 Genomics Research Center, Academia Sinica, Taipei, Taiwan; 5 Graduate Institute of Epidemiology and Preventative Medicine, College of Public Health, National Taiwan University, Taipei, Taiwan; 6 Institute of Clinical Medicine, National Yang-Ming University, Taipei, Taiwan; 7 Graduate Institute of Medicine, College of Medicine, Kaohsiung Medical University, Kaohsiung, Taiwan; 8 Biomedical Translation Research Center, Academia Sinica, Taipei, Taiwan; 9 Infections and Immunoepidemiology Branch, Division of Cancer Epidemiology and Genetics, National Institutes of Health, National Cancer Institute, 9609 Medical Center Dr, Rm 6-E212, Rockville, MD 20850 USA

**Keywords:** Epidemiology, Infectious diseases, Inflammation, Proteomics, Infectious diseases, Hepatitis, Liver diseases

## Abstract

Host immune response and chronic inflammation associated with chronic hepatitis B virus (HBV) infection play a key role in the pathogenesis of liver diseases such as cirrhosis and hepatocellular carcinoma (HCC). We sampled 175 HCC, 117 cirrhotic and 165 non-cirrhotic controls from a prospective cohort study of chronically HBV-infected individuals. Multivariable polytomous logistic regression and canonical discriminant analysis (CDA) were used to compare baseline plasma levels for 102 markers in individuals who developed cirrhosis vs. controls and those who developed HCC vs. cirrhosis. Leave-one-out cross validation was used to generate receiver operating characteristic curves to compare the predictive ability of marker groups. After multivariable adjustment, HGF (Q4v1OR: 3.74; p-trend = 0.0001), SLAMF1 (Q4v1OR: 4.07; p-trend = 0.0001), CSF1 (Q4v1OR: 3.00; p-trend = 0.002), uPA (Q4v1OR: 3.36; p-trend = 0.002), IL-8 (Q4v1OR: 2.83; p-trend = 0.004), and OPG (Q4v1OR: 2.44; p-trend = 0.005) were all found to be associated with cirrhosis development compared to controls; these markers predicted cirrhosis with 69% accuracy. CDA analysis identified a nine marker model capable of predicting cirrhosis development with 79% accuracy. No markers were significantly different between HCC and cirrhotic participants. In this study, we assessed immunologic markers in relation to liver disease in chronically-HBV infected individuals. While validation in required, these findings highlight the importance of immunologic processes in HBV-related cirrhosis.

## Introduction

The World Health Organization estimates that 257 million people worldwide are living with chronic hepatitis B virus (HBV) infection^1^. In spite of significant public health efforts over the past few decades, HBV-related liver cirrhosis and the increased risk of hepatocellular carcinoma (HCC) among infected persons remains a major health concern^2^. Liver disease etiology is heterogeneous. Nevertheless, the estimated fractions of cirrhosis and HCC attributable to HBV infection are believed to be as high as 30% and 53%, respectively, corresponding to over 200,000 annual deaths due to cirrhosis and 300,000 annual deaths due to HCC^3^.

Despite being at increased risk of liver disease, most individuals with HBV infection do not develop cirrhosis or HCC. The natural history of chronic HBV infection and subsequent disease, which remains to be fully elucidated, is impacted by both viral (HBV DNA levels, genotype and mutation patterns) and host-specific factors (age, sex, genetic polymorphisms, and immune status). Host immune response plays a critical role in the susceptibility to chronic HBV infection; 5–10% of adults with acute HBV infection fail to clear the virus and develop chronic disease due to inadequate adaptive immune response^4^. Studies on HBV pathogenesis in liver disease have largely been limited to experimental and small-scale clinical models, but consistently demonstrate the role of chronic inflammation in disease progression. A few studies have shown HBV specific T cells, chemokine-mediated neutrophil infiltrates, lymphocytes and natural killer cells to play a role in HBV-related liver damage^5,6^. This inflammatory microenvironment results in oxidative stress, ultimately promoting Kupffer cells to drive stellate activation through nuclear factor κB (NF-κB), leading to progressive fibrosis and ultimately cirrhosis^6–9^.

The role of circulating immunologic proteins have been investigated in prospective cohort studies in the context of HCC development, demonstrating the potential utility of markers such as C-reactive protein (CRP)^10,11^, interleukin-6 (IL-6)^11,12^, insulin-like growth factor binding protein-3 (IGFBP-3)^13,14^, and intercellular adhesion molecule 1 (ICAM-1)^15^ in predicting carcinogenesis. Nevertheless, no studies to our knowledge have comprehensively evaluated the role of circulating immunologic proteins on cirrhosis development in chronically HBV infected individuals. The morbidity and mortality associated with cirrhosis, and the accompanying risk of HCC among cirrhotic individuals are high. As such, assessing which circulating markers are associated with cirrhosis vs. chronic HBV infection and with HCC vs. cirrhosis may shed light and on the biological processes involved and potentially inform risk prediction. In this study, we utilize data from the Risk Evaluation of Viral Load Elevation and Associated Liver Disease/Cancer-Hepatitis B Virus (REVEAL-HBV) cohort to broadly evaluated the association between circulating immunologic markers and cirrhosis in chronically HBV infected participants.

## Methods

### Study population

REVEAL-HBV is a longitudinal, community-based cohort study that was developed to better understand the natural history and risk factors of HCC among chronic HBV carriers^4^. In brief, between 1991 and 1992, 23,820 individuals aged 30–65 years were recruited across seven townships in Taiwan. After serological assessment of baseline samples, 4155 participants were determined to be HBV surface antigen (HBsAg)-seropositive; among these individuals, 3653 were seronegative for hepatitis C antibodies with no evidence of HCC. Covariates such as age, HBV genotype, smoking, alcohol use, body mass index (BMI), and history of diabetes were collected. REVEAL-HBV was approved by the Institutional Review Board of the College of Public Health of the National Taiwan University (Taipei, Taiwan), all participants provided informed consent, and the study was conducted in accordance with all relevant guidelines and regulation.

Development of cirrhosis and HCC were both closely tracked within the REVEAL-HBV cohort through biannual or annual exams until December 31, 2008. Cirrhosis was detected using high-resolution, real-time abdominal ultrasound as well as a quantitative scoring system reflective of liver surface features (normal, irregular, undulated), liver parenchymal texture (normal, heterogeneous, coarse), size of the intrahepatic blood vessel (normal, obscure, narrowing) and splenic size (normal, enlarged). After 1999, cirrhosis was further diagnosed through data linkage with the National Taiwan Health Insurance Database and confirmed by medical chart review. HCC diagnosis was similarly assessed through follow-up examination using ultrasound, α-fetoprotein testing, or data linkage with the Taiwan National Cancer Registry. Additional linkage to the national death certificate program was conducted and incident cases of HCC were verified through medical review of histopathological records; HCC case verification was obtained when a lesion was detected using at least two imaging techniques (abdominal ultrasonography, angiogram, or computed tomography) and/or one imaging technique and a serum α-fetoprotein level of ≥ 400 ng/mL.

For this case–control analysis, 92 persons with cirrhosis and 129 chronic HBV carriers (i.e. controls) were matched to all diagnosed HCC cases on sex, age at sample collection, HBV DNA level (< 10,000 or ≥ 10,000 copies/mL), and time from entry to sample collection ± 2 years. In order to ensure the study was adequately powered, frequency matching on sex, age at sample collection and HBV DNA level (< 10,000 or ≥ 10,000 copies/mL) was used to identify an additional 25 persons with cirrhosis and 36 controls. At the time of sample selection cirrhosis was assessed through 2004 and HCC through 2011; controls were not required to survive until the end of follow-up.

### Marker measurements

Upon collection, all samples were stored at 70 °C at the Academia Sinica in Taipei, Taiwan, until 250 µL of heparin plasma were aliquoted onto 96 well plates and shipped to National Cancer Institute. Selected samples were from baseline or as near baseline as possible. 184 immunologic proteins were evaluated using the Olink (Uppsala, Sweden) inflammation and cardiometabolic panels, each panel requiring 1 μL of sample. The inflammation panel was designed in close collaboration with experts in inflammation-related diseases and includes a number of cytokines, chemokines, and other inflammation markers believed to be related to cancer; the cardiometabolic panel was selected because it includes the most liver-specific proteins. 48 blinded quality control samples from twelve patients with chronic HBV infection and twelve with chronic HCV infection were included on the panels. The limit of detection was achieved in > 90% of samples for all but 12 markers—which were excluded from analyses; an additional 69 markers were excluded due to intraclass correlation coefficients < 80% and one marker was removed due to a coefficient of variation > 25% (Supplementary Table S3). A total of 102 markers were included in the final analysis.

### Statistical analysis

Serological markers were evaluated as categorical variables and grouped in the following manner: for markers detectable in ≥ 75% of controls, quartiles were created based on values above the limit of detection (LLOD), with those at LLOD assigned to the lowest quartile. For markers detectable in 50–75% of controls, four categories were created with participants at LLOD being assigned to the lowest quartile, and the rest were divided into tertiles. For markers detectable in 25–50% of controls, tertiles were created with samples at LLOD comprising the lowest tertile and the remaining two categories calculated based on the median split value above LLOD. Finally, for markers detectable in < 25% of persons, binary coding was created based on those at vs. above LLOD.

Unconditional polytomous logistic regression models were used to assess the association between individual categorical circulating immunologic markers and stages of chronic HBV related liver disease, specifically: persons with cirrhosis compared to controls and HCC participants compared to persons with cirrhosis. The association between these markers and HCC vs. controls has been described previously^16^. Potential confounders were evaluated using a priori knowledge and backward selection. Final models included continuous age, sex, continuous years of follow-up, quartiles of HBV DNA (quartiles in controls: < 300–12,570, 12,571–45,919, 45,920–229,307, 229,308 + copies/mL), serum alanine aminotransferase (ALT) (< 15, 15–44, ≥ 45 U/L), alcohol, smoking, and HBV e-antigen (HBeAg). Given the large proportion of HCC cases with underlying cirrhosis, we conducted a sensitivity analysis for the associations between immunologic markers and HCC versus cirrhosis stratified by the presence of underlying cirrhosis in HCC cases. Statistical significance was determined after correcting for multiple testing using a Benjamini–Hochberg false discovery rate correction of 10%. To address potential concerns regarding differences in duration of infection, we conducted a sensitivity analysis stratified by HBV DNA level using median cut-offs among controls; additionally, we fit crude as well as age, sex, ALT, drinking, smoking and HBeAg adjusted linear regression models with marker levels as the outcome and tested an interaction between case status and viral load as the exposure. To assess the role of temporality on the association found between circulating markers and disease status, an additional sensitivity analysis stratified by median time to cirrhosis diagnosis among participants with cirrhosis who did not progress to HCC (6.57 years) was conducted. Statistical significance for these analyses was determined using an α = 0.05.

Canonical discriminant analysis (CDA) was used to discern linear groups of circulating immune markers found to be differentially expressed across the same comparison groups assessed by logistic regression models; statistical significance for backward selection was determined using an α = 0.05. Finally, leave-one-out cross validation^17^ was used to calculate the area under the receiver operating characteristic curves (AUCs) to evaluate and compare the predictive ability of three models: 1. clinical/demographic characteristics alone (age, sex, years of follow-up, HBV viral load, ALT, drinking, smoking, and HBeAg), 2. clinical/demographic variables plus the markers found to be significant in the logistic regression model, and 3. clinical/demographic variables plus the markers found to be significant in the CDA. Separate figures were generated for the comparison between persons who developed cirrhosis vs. controls and persons who developed HCC vs. cirrhosis only. Statistical significance for the comparison between the curves was determined based on the DeLong, DeLong, and Clarke-Pearson method using an α = 0.05. All statistical analyses were performed with SAS software version 9.4 (SAS Institute, Inc, Cary, NC).

## Results

The median time from sample collection to cirrhosis development among the 117 persons with cirrhosis was 6.57 years (range 0.36–11.98); the median time from sample collection to HCC diagnosis was 10.50 years (range 0.07–18.40). There were no major differences noted in terms of sex distribution, alcohol consumption, smoking, family history of HCC, or BMI across HCC cases, persons with cirrhosis and controls. HCC cases had higher baseline ALT levels and were more likely to be HBeAg positive compared to the other groups (Table 1). Approximately 62% (109/175) of participants who developed HCC had underlying cirrhosis.

**Table 1:** Participants characteristics.

Factor	REVEAL-HBV		
	HCC cases	Cirrhosis cases	Controls
Total N	175	117	165
% Male	80.6%	78.6%	81.2%
Median age at sample date (range)	51 (30–67)	46 (32–69)	51 (30–66)
Median year of serum collection (range)	1993 (1992–1997)	1993 (1992–1997)	1993 (1992–1998)
Median years of follow-up (range)*	15.4 (1.7–23.6)	19.8 (5.3–23.7)	19.9 (5.5–22.4)
Median BMI (range)	24.4 (17.0–37.7)	23.8 (16.4–32.6)	23.4 (17.2–33.4)
**ALT, N (%)**			
< 15	65 (37.1)	48 (41.0)	98 (59.4)
15–44	83 (47.4)	60 (51.3)	59 (35.8)
≥ 45	27 (15.4)	9 (7.7)	8 (4.8)
**Smoking**^**†**^			
No	103 (58.9)	71 (60.7)	108 (65.5)
Yes	71 (40.6)	46 (39.3)	57 (34.5)
**Alcohol drinking**^**†**^			
No	140 (80.0)	104 (88.9)	138 (83.6)
Yes	34 (19.4)	13 (11.1)	26 (15.8)
**Family history of HCC**			
No	158 (90.3)	112 (95.7)	151 (91.5)
Yes	17 (9.7)	5 (4.3)	14 (8.5)
**HBeAg positive at baseline**			
No	99 (56.6)	91 (77.8)	146 (88.5)
Yes	76 (43.4)	26 (22.2)	19 (11.5)
Median HBV DNA level (copies/ml) at sample date	1,938,060	141,426	45,920

*Time from entry to HCC diagnosis, death, or end of study (December 31, 2011), whichever came first.

^†^Numbers do not add to total due to missing values.

Assessment of individual markers using multivariable logistic regression yielded six proteins that were found to be positively associated with cirrhosis when compared to controls. Persons who developed cirrhosis were four times more likely to have elevated levels of signaling lymphocytic activation molecule 1 (SLAMF1) at baseline when compared to controls (odds ratio [OR]_Q4v.Q1_ = 4.07 [95% CI 1.85–8.99]); similar associations were found for hepatocyte growth factor (HGF) with an OR_Q4v.Q1_ = 3.74 (95% CI 1.67–8.38), colony stimulating factor 1 (CSF1) with an OR_Q4v.Q1_ = 3.00 (95% CI 1.38–6.49), urokinase-type plasminogen activator (uPA) with an OR_Q4v.Q1_ = 3.36 (95% CI 1.52–7.40), interleukin-8 (IL-8) with an OR_Q4v.Q1_ = 2.83 (95% CI 1.29–6.23), and OPG with an OR_Q4v.Q1_ = 2.44 (95% CI 1.14–5.22) (Table 2). We did not find any markers to be significantly associated with HCC risk when compared to persons with cirrhosis after FDR correction (Supplementary Table S1). These null results persisted in sensitivity analyses for the associations between immunologic markers and HCC versus cirrhosis stratified by the presence of underlying cirrhosis in HCC cases (Supplementary Table S2).

**Table 2:** Odds ratios* and 95% CIs for associations between selected markers and cirrhosis versus non-cirrhotic controls.

Analyte	Cirrhosis vs control				
	OR (95% CI)			p-trend	FDR-corrected p-trend
	Q2 v. Q1	Q3 v. Q1	Q4 v. Q1		
HGF	1.19 (0.49–2.91)	3.14 (1.37–7.21)	3.74 (1.67–8.38)	0.0001	0.007
SLAMF1	1.31 (0.56–3.06)	2.32 (1.01–5.35)	4.07 (1.85–8.99)	0.0001	0.007
CSF1	1.42 (0.61–3.28)	3.05 (1.36–6.82)	3.00 (1.38–6.49)	0.002	0.05
uPA	1.69 (0.72–3.96)	2.14 (0.94–4.85)	3.36 (1.52–7.40)	0.002	0.05
IL8	1.70 (0.76–3.84)	2.95 (1.36–6.38)	2.83 (1.29–6.23)	0.004	0.08
OPG	0.80 (0.34–1.87)	1.98 (0.94–4.21)	2.44 (1.14–5.22)	0.005	0.08

*Adjusted for age, sex, years of follow up, HBV viral load, serum alanine aminotransferase (ALT) level, alcohol, smoking, and HBV e antigen (HBeAg).

The sensitivity analysis stratified by HBV DNA level at baseline yielded similar results as those reported in the primary analysis. All six markers were found to be significantly associated with cirrhosis among those with high (≥ 45,920 copies/mL) viral levels. Among those with low HBV viral levels at baseline (< 45,920 copies/mL) only the associations between HGF, SLAMF1, uPA, and cirrhosis were found to be statistically significant (Table 3). We did not find evidence for a numerical relationship between these markers and HBV DNA level in linear regression models (data not shown). In assessing whether temporality plays a role in the observed associations, all six markers were significantly associated with cirrhosis development among those diagnosed < 6.57 years after serum collection. Additionally, the effect sizes for these markers were larger within this subset when compared to the original analysis. HGF, SLAMF1, CSF1, and uPA were associated with cirrhosis among those diagnosed ≥ 6.57 years after serum collection (Table 4).

**Table 3:** REVEAL-HBV odds ratios* and 95%CIs stratified by viral load.

Analyte	Low HBV viral level (< 45,920)				High HBV viral level (≥ 45,920)			
	p-trend	OR (95% CI)			p-trend	OR (95% CI)		
		Q2 v. Q1	Q3 v. Q1	Q4 v. Q1		Q2 v. Q1	Q3 v. Q1	Q4 v. Q1
HGF	0.03	1.85 (0.45–7.65)	3.25 (0.79–13.35)	4.26 (1.09–16.58)	0.004	0.99 (0.30–3.27)	2.97 (1.02–8.69)	3.40 (1.22–9.52)
SLAMF1	0.02	4.91 (1.07–22.48)	3.56 (0.77–16.5)	6.80 (1.56–29.59)	0.003	0.61 (0.20–1.84)	2.26 (0.76–6.74)	3.03 (1.12–8.21)
CSF1	0.36	1.66 (0.45–6.14)	3.21 (0.85–12.12)	1.64 (0.43–6.23)	0.002	1.23 (0.40–3.78)	2.62 (0.93–7.40)	3.97 (1.48–10.67)
uPA	0.04	2.74 (0.58–12.94)	2.77 (0.59–12.94)	4.87 (1.10–21.65)	0.04	1.75 (0.59–5.21)	1.92 (0.70–5.28)	2.97 (1.11–7.99)
IL8	0.41	1.45 (0.38–5.62)	1.89 (0.54–6.61)	1.74 (0.45–6.72)	0.01	1.54 (0.54–4.40)	3.64 (1.30–10.20)	3.03 (1.11–8.22)
OPG	0.09	1.29 (0.34–4.86)	2.05 (0.6–7.02)	2.71 (0.75–9.8)	0.03	0.53 (0.17–1.68)	2.08 (0.77–5.63)	2.24 (0.83–6.02)

*Adjusted for age, sex, years of follow up, HBV viral load, serum alanine aminotransferase (ALT) level, alcohol, smoking, and HBV e antigen (HBeAg).

**Table 4:** REVEAL-HBV odds ratios* and 95% CIs stratified by time to cirrhosis diagnosis.

Analyte	Early cirrhosis diagnosis (< 6.57 years)				Late cirrhosis diagnosis (≥ 6.57 years)			
	p-trend	OR (95% CI)			p-trend	OR (95% CI)		
		Q2 v. Q1	Q3 v. Q1	Q4 v. Q1		Q2 v. Q1	Q3 v. Q1	Q4 v. Q1
HGF	0.0006	0.88 (0.23, 3.36)	2.95 (0.94, 9.3)	4.48 (1.51, 13.27)	0.007	1.56 (0.54, 4.51)	3.56 (1.31, 9.69)	3.32 (1.21, 9.09)
SLAMF1	0.001	2.67 (0.74, 9.67)	3.32 (0.92, 11.97)	6.83 (2.01, 23.22)	0.003	0.78 (0.28, 2.23)	1.96 (0.74, 5.22)	3.14 (1.26, 7.81)
CSF1	0.0008	2.50 (0.69, 9.05)	6.37 (1.91, 21.27)	6.20 (1.90, 20.26)	0.04	1.09 (0.41, 2.87)	1.90 (0.74, 4.90)	2.21 (0.91, 5.39)
uPA	0.010	2.18 (0.70, 6.80)	1.04 (0.30, 3.56)	4.10 (1.45, 11.55)	0.02	1.44 (0.50, 4.15)	3.06 (1.16, 8.07)	2.60 (0.97, 6.97)
IL8	0.006	1.24 (0.41, 3.72)	2.79 (1.04, 7.47)	3.37 (1.22, 9.32)	0.05	2.15 (0.78, 5.90)	3.33 (1.25, 8.86)	2.48 (0.91, 6.74)
OPG	0.0008	0.41 (0.09, 1.84)	2.69 (0.93, 7.74)	4.09 (1.44, 11.64)	0.20	0.95 (0.37, 2.44)	1.64 (0.68, 4.00)	1.60 (0.63, 4.02)

*Adjusted for age, sex, years of follow up, HBV viral load, serum alanine aminotransferase (ALT) level, alcohol, smoking, and HBV e antigen (HBeAg).

**Figure 1:**
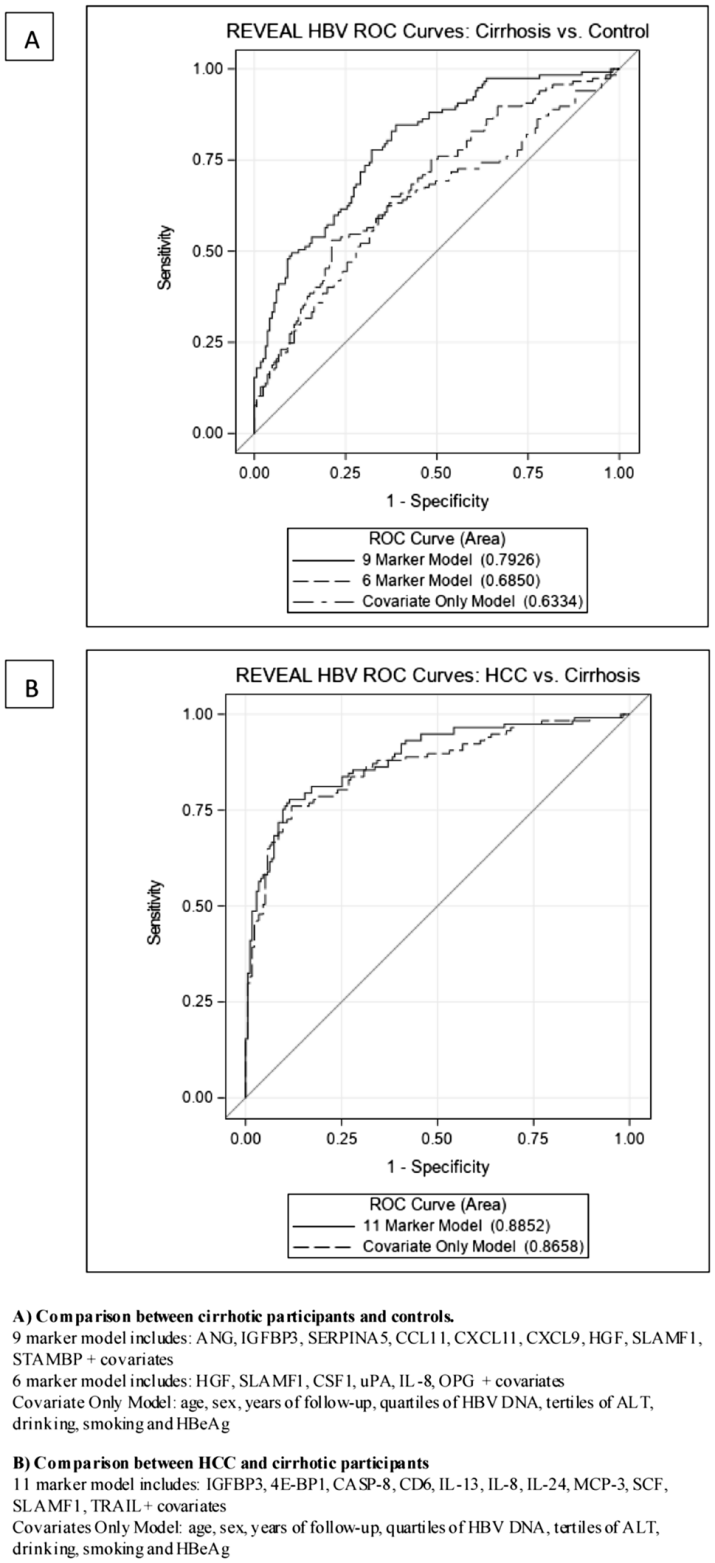
Receiver operating characteristic (ROC) curves comparing predictive models.

CDA isolated nine markers that together differentiated between those who developed cirrhosis vs. controls; these markers included HGF and SLAMF1, as well as angiogenin (ANG), insulin-like growth factor-binding protein 3 (IGFBP3), plasma serine protease inhibitor (SERPINA5), eotaxin (CCL11), two C-X-C motif chemokines (CXCL11 and CXCL9), and STAM-binding protein (STAMBP). ROC curves for this comparison grouping yielded an area under the curve (AUC) of 63.3% for the clinical/demographic variables only model. ROC curves for the six markers identified in the logistic regression model in addition to the clinical/demographic variables improved predictability to 68.5%. Lastly, the nine marker model from the CDA analysis in addition to the clinical/demographic variables yielded an AUC of 79.3% (Fig. 1); ROC contrast test results found AUC curves for the marker inclusive models to significantly improved predictability when compared to the clinical/demographic only model, with a p-value ≤ 0.0001.

CDA analysis for the comparison between those who developed HCC vs. those who developed cirrhosis but not HCC yielded 11 markers that together were found to be significantly different between these groups, including: IGFBP3, SLAMF1, eukaryotic translation initiation factor 4E-binding protein 1 (4E-BP1), caspase-8 (CASP-8), T cell surface glycoprotein CD6 isoform (CD6), three interleukin (IL-8, IL-13, IL-24), monocyte chemotactic protein 3 (MCP-3), stem cell factor (SCF), and TNF-related apoptosis-inducing ligand (TRAIL). Due to the lack of significant markers found in the logistic regression analysis for this comparison, only two ROC curves were generated; no significant difference (p-value = 0.07) was found between the clinical/demographic variable only model (AUC = 86.6%) and the model that included the 11 markers from the CDA analysis (AUC = 88.5%) (Fig. 1).

## Discussion

This is the first study to our knowledge to evaluate a broad list of immunologic markers as they relate to the development of cirrhosis in a prospective observational cohort of HBV infected participants. Herein, we identified six individual proteins (HGF, SLAMF, CSF1, uPA, IL-8, OPG) to be significantly positively associated with the development of cirrhosis; these markers were found to be particularly strongly associated among individuals who developed cirrhosis within seven years after sample collection. Lack of an association between these immunologic markers and HCC suggests that these markers may influence progression to cirrhosis among HBV infected individuals, but not further progression to HCC. Compared to clinical/demographic variables alone, the addition of the six markers improved predictability of cirrhosis development by about 6% (63% vs. 69%). In addition to evaluating the markers individually, we assessed how well a combination of markers might discriminate between individuals with chronic HBV infection who go on to develop cirrhosis versus those who do not. This analysis identified a nine-protein set comprised of ANG, IGFBP3, SERPINA5, CCL11, CXCL11, CXCL9, HGF, SLAMF1, and STAMBP that in addition to clinical/demographic variables was able to predict cirrhosis development with 79% accuracy in a cross-validation model.

Our study found increased levels of HGF, CSF1, and uPA—three markers believed to be associated with liver regeneration—to be associated with greater odds of developing clinical cirrhosis. HGF, which is primarily secreted by mesenchymal cells, functions to regulate cellular motility and development and has previously been shown to be elevated in cirrhotic patients^18,19^. The prevailing hypothesis is that fibroblasts drive the increased production HGF to stimulate growth of Met-positive hepatocytes. In the context of repeated injury due to an infectious agent such as HBV, continued regeneration of liver cells can result in the overgrowth of fibroblasts that ultimately result in the development of cirrhosis^20^. Albeit through a different method, CSF1 controls macrophage numbers that facilitate the hepatic innate immune defense and ultimately support hepatocyte proliferation following injury. In transplant and donor patients, circulating CSF1 has been shown to be elevated in association with rapid liver regrowth^21,22^. Similarly, CSF1 has also been reported to be elevated in patients with acute liver injury as well as those diagnosed with cirrhosis and HCC^22^. Finally, uPA is a serine protease that has been implicated in the direct and indirect degradation of matrix proteins and is believed to have a bidirectional effect in the liver. Increased expression of the uPA cell-surface binding protein results in increased hepatic uPA in the early stages of liver regeneration, nonetheless in later phases, uPA derived plasmin leads to the suppression of hepatocyte growth^23–25^. While uPA has previously been reported to be elevated in fibrotic/cirrhotic patients^26^, its dual effects on hepatocytes has recently spurred interest in its utility in gene therapy^27,28^.

In accordance with HBV mediated inflammation, we found elevated levels of IL-8 and SLAMF1 to be associated with the development of clinical cirrhosis. IL-8 is a CXC chemokine that has been well established as a major factor in acute inflammation. Serum IL-8 levels have been associated with disease progression in chronically infected hepatitis C virus (HCV) and HBV patients^29,30^. Increased levels of IL-8 in both the liver and in circulation have been reported in alcoholic hepatitis, ischemia–reperfusion injury, and fibrosis/cirrhosis^31–34^. Similarly, CXCL11 and CXCL9 were only marginally significant after FDR correction in logistic regression analyses but were both identified as differentiating markers of cirrhosis in CDA analyses. Elevated levels of these proteins, as seen in our analysis, have previously been reported in liver diseases (both with and without infectious etiology) and functionally linked to inflammation, hepatic injury and fibrosis^35–37^. SLAMF1 (recently renamed CD150) is an endoglin that modulates TGF- β signaling and has been proposed to function in regulating the epithelial-mesenchymal transition associated with tissue repair, fibrogenesis and carcinogenesis^38–40^. Elevated levels of SLAMF1 have been associated with TGF- β1 upregulation, inflammation and more recently cirrhosis^41^.

Cirrhosis can be associated with a number of metabolic disorders including osteopenia and osteoporosis, insulin resistance, and malnutrition^42–44^. Receptor activator of nuclear factor κB ligand (RANKL) and OPG work together to maintain bone homeostasis by binding to the receptor RANK. Together, the OPG/RANKL system plays a role in linking the immune system and bone metabolism. Previous studies have demonstrated high levels of OPG and low levels of RANKL in cirrhotic patients compared to those with non-cirrhotic chronic liver disease and control populations^45^; this phenomenon is believed to be due to a compensatory response to halt bone loss within affected persons^46^. In accordance with this literature, but for the first time in chronically infected HBV participants, we found elevated levels of OPG to be associated with increased odds of cirrhosis development. In contrast, lower levels of IGFBP3 were associated with cirrhosis development. In CDA analysis, IGFBP-3 was determined to differentiate persons with cirrhosis from controls; decreased levels of IGFBP-3 in persons with cirrhosis have previously been described and attributed to the fact that IGFBP-3 is predominantly synthesized by hepatocytes and reportedly associated with severity of liver dysfunction^47,48^.

A major strength of the study was that REVEAL-HBV is a well characterized prospective cohort study that provides a unique opportunity to evaluate the association between immunologic markers and development of cirrhosis in chronically HBV infected individuals; nevertheless, this study does have several limitations. There is a possibility for misclassification bias; because cirrhosis was not diagnosed using data linkage with the National Taiwan Health Insurance Database until after 1999, under-diagnosis of cirrhosis from 1991 to 1999 is possible. However, cohort participants received an abdominal ultrasonography every 6–12 months, substantially reducing the risk of under-diagnosis even without linkage to the insurance database. Additionally, we did not have data on the use of anti-HBV therapies, which could potentially mask associations; however, antiviral therapy was not reimbursed by the Taiwanese universal health care system through 2003, which encompasses most of the study period, and after 2003 was only provided to high-risk patients under stringent criteria. Finally, due to the limited sample size, analyses between serum markers and disease may have been underpowered in stratified analyses.

Better understanding the natural history of disease among high risk patients with chronic HBV may offer important insights into prevention and treatment of liver disease. In this novel, comprehensive evaluation of circulating immunologic proteins, we identified several markers that were found to significantly distinguish and predict cirrhosis development, particularly among those diagnosed within 7 years of blood draw. While screening protocols for patients chronically infected with HBV do exist, the addition of serum biomarkers may aid in risk stratification to further enhance these procedures. Although this study requires replication, these findings highlight the importance of immunologic and proliferative pathways in HBV-related liver disease.

## Supplementary Information

Supplementary Information.

## References

[1] World Health Organization (2017). Global Hepatitis Report 2017.

[2] European Association for the Study of the Liver (2017). EASL 2017 Clinical practice guidelines on the management of hepatitis B virus infection. J. Hepatol..

[3] Perz JF, Armstrong GL, Farrington LA, Hutin YJ, Bell BP (2006). The contributions of hepatitis B virus and hepatitis C virus infections to cirrhosis and primary liver cancer worldwide. J. Hepatol..

[4] Chen CJ, Yang HI (2011). Natural history of chronic hepatitis B REVEALed. J. Gastroenterol. Hepatol..

[5] Edmunds W, Medley G, Nokes D, Hall A, Whittle H (1993). The influence of age on the development of the hepatitis B carrier state. Proc. R. Soc. Lond. B.

[6] Suhail M (2014). Potential mechanisms of hepatitis B virus induced liver injury. World J. Gastroenterol..

[7] Ganem D, Prince AM (2004). Hepatitis B virus infection—Natural history and clinical consequences. N. Engl. J. Med..

[8] Fattovich G, Stroffolini T, Zagni I, Donato F (2004). Hepatocellular carcinoma in cirrhosis: Incidence and risk factors. Gastroenterology.

[9] Luedde T, Schwabe RF (2011). NF-κB in the liver—linking injury, fibrosis and hepatocellular carcinoma. Nat. Rev. Gastroenterol. Hepatol..

[10] Chen W (2015). Association between C-reactive protein, incident liver cancer, and chronic liver disease mortality in the Linxian Nutrition Intervention Trials: A nested case–control study. Cancer Epidemiol. Prev. Biomark..

[11] Aleksandrova K (2014). Inflammatory and metabolic biomarkers and risk of liver and biliary tract cancer. Hepatology.

[12] Ohishi W (2014). Serum interleukin-6 associated with hepatocellular carcinoma risk: A nested case–control study. Int. J. Cancer.

[13] Adachi Y (2016). Insulin-like growth factor-related components and the risk of liver cancer in a nested case-control study. Tumor Biol..

[14] Major J (2010). Insulin-like growth factors and liver cancer risk in male smokers. Br. J. Cancer.

[15] Chen VL (2017). Soluble intercellular adhesion molecule-1 is associated with hepatocellular carcinoma risk: Multiplex analysis of serum markers. Sci. Rep..

[16] Koshiol J (2021). Immunologic markers and risk of hepatocellular carcinoma in hepatitis B virus‐and hepatitis C virus‐infected individuals. Aliment. Pharmacol. & Therap..

[17] Molinaro AM, Simon R, Pfeiffer RM (2005). Prediction error estimation: A comparison of resampling methods. Bioinformatics.

[18] Costantini S (2013). Cancer biomarker profiling in patients with chronic hepatitis C virus, liver cirrhosis and hepatocellular carcinoma. Oncol. Rep..

[19] Costantini S (2010). Serum cytokine levels as putative prognostic markers in the progression of chronic HCV hepatitis to cirrhosis. Eur. Cytokine Netw..

[20] Xie Q (2013). Overexpression of HGF promotes HBV-induced hepatocellular carcinoma progression and is an effective indicator for Met-targeting therapy. Genes Cancer.

[21] Matsumoto K (2013). Serial changes of serum growth factor levels and liver regeneration after partial hepatectomy in healthy humans. Int. J. Mol. Sci..

[22] Stutchfield BM (2015). CSF1 restores innate immunity after liver injury in mice and serum levels indicate outcomes of patients with acute liver failure. Gastroenterology.

[23] Andreasen PA, Kjøller L, Christensen L, Duffy MJ (1997). The urokinase-type plasminogen activator system in cancer metastasis: A review. Int. J. Cancer.

[24] De Petro G (1998). Expression of urokinase-type plasminogen activator (u-PA), u-PA receptor, and tissue-type PA messenger RNAs in human hepatocellular carcinoma. Cancer Res..

[25] Chan C-F (2004). Evaluation of nuclear factor-κB, urokinase-type plasminogen activator, and HBx and their clinicopathological significance in hepatocellular carcinoma. Clin. Cancer Res..

[26] Arbabi Bidgoli S, Djamali Zaverhei M, Mohagheghi M (2007). Differential expression of uPA in chronic hepatitis B and C, liver cirrhosis and hepatocellular carcinoma: Comparison with normal liver tissues and liver metastatic tumors. Int. J. Cancer Res..

[27] Salgado S (2000). Liver cirrhosis is reverted by urokinase-type plasminogen activator gene therapy. Mol. Ther..

[28] Iredale JPJG (2004). A cut above the rest? MMP-8 and liver fibrosis gene therapy. Gastroenterology.

[29] Polyak SJ, Khabar KS, Rezeiq M, Gretch DR (2001). Elevated levels of interleukin-8 in serum are associated with hepatitis C virus infection and resistance to interferon therapy. J. Virol..

[30] Yang K (2014). Enhanced levels of interleukin-8 are associated with hepatitis B virus infection and resistance to interferon-alpha therapy. Int. J. Mol. Sci..

[31] Jaeschke H (2006). Mechanisms of liver injury. II. Mechanisms of neutrophil-induced liver cell injury during hepatic ischemia-reperfusion and other acute inflammatory conditions. Am. J. Physiol. Gastrointest. Liver Physiol..

[32] Jaeschke H (2002). Neutrophil-mediated tissue injury in alcoholic hepatitis. Alcohol.

[33] Dominguez M (2009). Hepatic expression of CXC chemokines predicts portal hypertension and survival in patients with alcoholic hepatitis. Gastroenterology.

[34] Zimmermann HW (2011). Interleukin-8 is activated in patients with chronic liver diseases and associated with hepatic macrophage accumulation in human liver fibrosis. PLoS ONE.

[35] Larrubia JR, Benito-Martinez S, Calvino M, Sanz-de-Villalobos E, Parra-Cid T (2008). Role of chemokines and their receptors in viral persistence and liver damage during chronic hepatitis C virus infection. World J. Gastroenterol..

[36] Zeremski M (2008). Intrahepatic levels of CXCR3-associated chemokines correlate with liver inflammation and fibrosis in chronic hepatitis C. Hepatology.

[37] Apolinario A (2002). Increased expression of T cell chemokines and their receptors in chronic hepatitis C: Relationship with the histological activity of liver disease. Am. J. Gastroenterol..

[38] Barbara NP, Wrana JL, Letarte MJ (1999). Endoglin is an accessory protein that interacts with the signaling receptor complex of multiple members of the transforming growth factor-β superfamily. J. Biol. Chem..

[39] Li C (2000). CD105 antagonizes the inhibitory signaling of transforming growth factor βl on human vascular endothelial cells. FASEB J..

[40] Li C (2003). TNFα down-regulates CD105 expression in vascular endothelial cells: A comparative study with TGFß1. Anticancer Res..

[41] Yagmur E (2007). Elevation of endoglin (CD105) concentrations in serum of patients with liver cirrhosis and carcinoma. Eur. J. Gastroenterol. Hepatol..

[42] Leslie WD, Bernstein CN, Leboff MS (2003). AGA technical review on osteoporosis in hepatic disorders. Gastroenterology.

[43] Huisman EJ, Trip EJ, Siersema PD, van Hoek B, van Erpecum KJ (2011). Protein energy malnutrition predicts complications in liver cirrhosis. Eur. J. Gastroenterol. Hepatol..

[44] Goral V, Atalay R, Kucukoner M (2010). Insulin resistance in liver cirrhosis. Hepatogastroenterology.

[45] Moschen AR (2005). The RANKL/OPG system and bone mineral density in patients with chronic liver disease. J. Hepatol..

[46] López-Larramona G, Lucendo AJ, González-Castillo S, Tenias JM (2011). Hepatic osteodystrophy: An important matter for consideration in chronic liver disease. World J. Hepatol..

[47] Nedic O, Nikolic JA, Prisic S, Acimovic J, Hajdukovic-Dragojlovic L (2003). Reactivity of IGF binding protein-3 isoforms towards concanavalin A in healthy adults and subjects with cirrhosis. Addict. Biol..

[48] Wu YL, Ye J, Zhang S, Zhong J, Xi RP (2004). Clinical significance of serum IGF-I, IGF-II and IGFBP-3 in liver cirrhosis. World J. Gastroenterol..

