# Supplementary material for: Association between immunologic markers and cirrhosis in individuals with chronic hepatitis B
Source: Sci Rep. 2021 Nov 15;11:21194. doi: 10.1038/s41598-021-00455-8 (PMC8593047; doi:10.1038/s41598-021-00455-8)
Supplement: Supplementary file 1 — Supplementary Information. [file 41598_2021_455_MOESM1_ESM.docx]

**Supplementary Table 1:** Odds ratios* and 95% CIs for associations between selected markers and HCC versus cirrhosis.

| **Analyte** | **HCC vs. Cirrhosis** | | | | |  |
| --- | --- | --- | --- | --- | --- | --- |
|  |  | **OR (95% CI)** |  | **P-trend** | **FDR-corrected**  **P-trend** |  |
|  | **Q2 v. Q1** | **Q3 v. Q1** | **Q4 v. Q1** |  |  |  |
| HGF | 1.58(0.49,5.06) | 0.96 (0.34,2.73) | 0.63 (0.23,1.75) | 0.12 | 0.50 |  |
| SLAMF1 | 0.91(0.33,2.49) | 0.47 (0.17,1.29) | 0.40 (0.16,1.01) | 0.02 | 0.46 |  |
| CSF1 | 0.84(0.3,2.34) | 0.71 (0.28,1.80) | 0.77 (0.31,1.88) | 0.57 | 0.80 |  |
| uPA | 0.52(0.19,1.47) | 0.66 (0.26,1.70) | 0.41 (0.17,0.98) | 0.07 | 0.46 |  |
| IL8 | 1.05(0.39,2.82) | 0.80 (0.33,1.96) | 0.65 (0.25,1.67) | 0.28 | 0.67 |  |
| OPG | 1.04(0.37,2.89) | 0.54 (0.22,1.34) | 0.51 (0.21,1.23) | 0.07 | 0.46 |  |
| *Adjusted for age, sex, years of follow up, HBV viral load, serum alanine aminotransferase (ALT) level, alcohol, smoking, and HBV e antigen (HBeAg) | | | | | | |

**Supplementary Table 2:** Odds ratios* and 95%CIs for associations between selected markers and hepatocellular carcinoma (HCC) versus cirrhosis stratified by the presence of underlying cirrhosis in HCC cases

| **Analyte** | **Non-cirrhotic HCC cases vs. Cirrhosis** ꭞ | | | | |  | **Cirrhotic HCC cases vs. Cirrhosis^‡^** | | | | |
| --- | --- | --- | --- | --- | --- | --- | --- | --- | --- | --- | --- |
|  | **P-trend** | **FDR** |  | **OR (95% CI)** |  |  | **P-trend** | **FDR** |  | **OR (95% CI)** |  |
|  |  |  | **Q2 v. Q1** | **Q3 v. Q1** | **Q4 v. Q1** |  |  |  | **Q2 v. Q1** | **Q3 v. Q1** | **Q4 v. Q1** |
| HGF | 0.04 | 0.50 | 1.24(0.33,4.65) | 0.66(0.2,2.2) | 0.39(0.12,1.29) |  | 0.29 | 0.62 | 1.87 (0.43,8.08) | 1.17 (0.31,4.40) | 1.78 (0.21,2.83) |
| SLAMF1 | 0.03 | 0.40 | 1.05(0.33,3.36) | 0.48(0.15,1.56) | 0.4(0.13,1.18) |  | 0.04 | 0.48 | 0.58 (0.17,1.98) | 0.37 (0.11,1.23) | 0.32 (0.10,0.96) |
| CSF1 | 0.40 | 0.79 | 1.09(0.33,3.62) | 0.89(0.29,2.69) | 0.69(0.23,2.05) |  | 0.61 | 0.84 | 0.53 (0.15,1.83) | 0.47 (0.15,1.49) | 0.67 (0.24,1.90) |
| uPA | 0.006 | 0.27 | 0.42(0.12,1.42) | 0.75(0.26,2.14) | 0.18(0.06,0.55) |  | 0.42 | 0.70 | 0.50 (0.14,1.77) | 0.41 (0.12,1.39) | 0.57 (0.21,1.60) |
| IL8 | 0.79 | 0.79 | 0.98(0.29,3.27) | 0.73(0.25,2.18) | 0.7(0.23,2.15) |  | 0.34 | 0.65 | 1.20 (0.37,3.92) | 0.77 (0.26,2.26) | 0.68 (0.22,2.15) |
| OPG | 0.01 | 0.27 | 0.96(0.3,3.06) | 0.44(0.15,1.29) | 0.32(0.11,0.9) |  | 0.39 | 0.69 | 0.88 (0.24,3.25) | 0.62 (0.20,1.92) | 0.65 (0.22,1.89) |
| ꭞ N (cirrhosis/HCC): 117/66 **^‡^** N (cirrhosis/HCC): 117/109  *All models adjusted for age, sex, years of follow up, HBV viral load, serum alanine aminotransferase (ALT) level, alcohol, smoking, and HBV e antigen (HBeAg) | | | | | | | | | | | |

**Supplementary Table 3:** Markers tested in REVEAL-HBV; coefficients of variation (CV) and intraclass correlation coefficients (ICC) for markers with >90% detection among hepatocellular carcinoma (HCC) cases, cirrhosis cases, or non-cirrhosis controls; and reason for exclusion if excluded from analysis.

| **Protein** | **ID** | **Panel** | **CV** | **ICC** | **Reason if excluded** |
| --- | --- | --- | --- | --- | --- |
| Adenosine Deaminase (ADA) | P00813 | Inflammation | 7.43 | 89.44 |  |
| Angiogenin (ANG) | P03950 | Cardiometabolic | 4.27 | 83.6 |  |
| Angiopoietin-related protein 3 (ANGPTL3) | Q9Y5C1 | Cardiometabolic | 16.8 | 63.3 | ICC<80% |
| Apolipoprotein M (APOM) | O95445 | Cardiometabolic | 14.99 | 58.57 | ICC<80% |
| Artemin (ARTN)* | Q5T4W7 | Inflammation | 12.42 |  |  |
| Axin-1 (AXIN1) | O15169 | Inflammation | 4.21 | 98.99 |  |
| Beta-Ala-His dipeptidase (CNDP1) | Q96KN2 | Cardiometabolic | 23.86 | 68.89 | ICC<80% |
| Beta-galactoside alpha-2,6-sialyltransferase 1 (ST6GAL1) | P15907 | Cardiometabolic | 13.11 | 57.28 | ICC<80% |
| Beta-nerve growth factor (Beta-NGF) | P01138 | Inflammation | 2.27 | 86.64 |  |
| Cadherin-1 (CDH1) | P12830 | Cardiometabolic | 7.73 | 59.75 | ICC<80% |
| Carbonic anhydrase 1 (CA1) | P00915 | Cardiometabolic | 5.31 | 94.4 |  |
| Carbonic anhydrase 3 (CA3) | P07451 | Cardiometabolic | 34.76 | 98.51 | CV>25% |
| Carbonic anhydrase 4 (CA4) | P22748 | Cardiometabolic | 33.75 | 47.39 | ICC<80% |
| Cartilage acidic protein 1 (CRTAC1) | Q9NQ79 | Cardiometabolic | 19.28 | 67.4 | ICC<80% |
| Cartilage oligomeric matrix protein (COMP) | P49747 | Cardiometabolic | 3.9 | 82.82 |  |
| Caspase-8 (CASP-8 ) | Q14790 | Inflammation | 2.24 | 99.33 |  |
| C-C motif chemokine 14 (CCL14) | Q16627 | Cardiometabolic | 4.66 | 74.5 | ICC<80% |
| C-C motif chemokine 18 (CCL18) | P55774 | Cardiometabolic | 5.58 | 90.63 |  |
| C-C motif chemokine 19 (CCL19) | Q99731 | Inflammation | 1.83 | 97.97 |  |
| C-C motif chemokine 20 (CCL20) | P78556 | Inflammation | 2.58 | 98.8 |  |
| C-C motif chemokine 23 (CCL23) | P55773 | Inflammation | 2.16 | 89.35 |  |
| C-C motif chemokine 25 (CCL25) | O15444 | Inflammation | 3.1 | 96.86 |  |
| C-C motif chemokine 28 (CCL28) | Q9NRJ3 | Inflammation | 5.93 | 95.82 |  |
| C-C motif chemokine 3 (CCL3) | P10147 | Inflammation | 2.57 | 99.19 |  |
| C-C motif chemokine 4 (CCL4 ) | P13236 | Inflammation | 2.34 | 98.34 |  |
| C-C motif chemokine 5 (CCL5) | P13501 | Cardiometabolic | 10.54 | 97.23 |  |
| CD40L receptor (CD40) | P25942 | Inflammation | 1.28 | 97.12 |  |
| CD59 glycoprotein (CD59) | P13987 | Cardiometabolic | 23.68 | 40.88 | ICC<80% |
| Coagulation factor VII (F7) | P08709 | Cardiometabolic | 5.9 | 74.48 | ICC<80% |
| Coagulation factor XI (F11) | P03951 | Cardiometabolic | 3.67 | 72.52 | ICC<80% |
| Collagen alpha-1(XVIII) chain (COL18A1) | P39060 | Cardiometabolic | 10.97 | 77.51 | ICC<80% |
| Complement C1q tumor necrosis factor-related protein 1 (C1QTNF1) | Q9BXJ1 | Cardiometabolic | 14.02 | 68.93 | ICC<80% |
| Complement C2 (C2) | P06681 | Cardiometabolic | 5.88 | 83.68 |  |
| Complement factor H-related protein 5 (CFHR5) | Q9BXR6 | Cardiometabolic | 5.16 | 88.81 |  |
| Complement receptor type 2 (CR2) | P20023 | Cardiometabolic | 4.96 | 90.57 |  |
| CUB domain-containing protein 1 (CDCP1) | Q9H5V8 | Inflammation | 4.5 | 97.44 |  |
| C-X-C motif chemokine 1 (CXCL1) | P09341 | Inflammation | 1.42 | 98.71 |  |
| C-X-C motif chemokine 10 (CXCL10 ) | P02778 | Inflammation | 2.16 | 97.99 |  |
| C-X-C motif chemokine 11 (CXCL11) | O14625 | Inflammation | 1.6 | 95.74 |  |
| C-X-C motif chemokine 5 (CXCL5 ) | P42830 | Inflammation | 1.58 | 99.26 |  |
| C-X-C motif chemokine 6 (CXCL6) | P80162 | Inflammation | 2.01 | 97.27 |  |
| C-X-C motif chemokine 9 (CXCL9 ) | Q07325 | Inflammation | 2.14 | 96.28 |  |
| Cystatin D (CST5) | P28325 | Inflammation | 1.74 | 97.69 |  |
| Cystatin-C (CST3) | P01034 | Cardiometabolic | 4.25 | 73.52 | ICC<80% |
| Delta and Notch-like epidermal growth factor-related receptor (DNER) | Q8NFT8 | Inflammation | 1.4 | 83.45 |  |
| Dipeptidyl peptidase 4 (DPP4) | P27487 | Cardiometabolic | 8.3 | 66.84 | ICC<80% |
| EGF-containing fibulin-like extracellular matrix protein 1 (EFEMP1) | Q12805 | Cardiometabolic | 7.15 | 72.37 | ICC<80% |
| Endoglin (ENG) | P17813 | Cardiometabolic | 21.02 | 46.99 | ICC<80% |
| Eotaxin (CCL11) | P51671 | Inflammation | 0.81 | 90.05 |  |
| Eukaryotic translation initiation factor 4E-binding protein 1 (4E-BP1) | Q13541 | Inflammation | 4.41 | 97.57 |  |
| Fetuin-B (FETUB) | Q9UGM5 | Cardiometabolic | 24.77 | 72.36 | ICC<80% |
| Fibroblast growth factor 19 (FGF-19) | O95750 | Inflammation | 2.15 | 98.09 |  |
| Fibroblast growth factor 21 (FGF-21) | Q9NSA1 | Inflammation | 3.76 | 99.06 |  |
| Fibroblast growth factor 23 (FGF-23) | Q9GZV9 | Inflammation | 5.19 | 94.46 |  |
| Fibroblast growth factor 5 (FGF-5) | Q8NF90 | Inflammation |  |  | Low detection |
| Ficolin-2 (FCN2) | Q15485 | Cardiometabolic | 13.72 | 79.61 | ICC<80% |
| Fms-related tyrosine kinase 3 ligand (Flt3L) | P49771 | Inflammation | 1.86 | 92.17 |  |
| Fractalkine (CX3CL1 ) | P78423 | Inflammation | 2.73 | 91.27 |  |
| Glial cell line-derived neurotrophic factor (GDNF) | P39905 | Inflammation | 8.04 | 73.13 | ICC<80% |
| Glutaminyl-peptide cyclotransferase (QPCT) | Q16769 | Cardiometabolic | 9.07 | 69.86 | ICC<80% |
| Granulysin (GNLY) | P22749 | Cardiometabolic | 18.65 | 68.82 | ICC<80% |
| Growth arrest-specific protein 6 (GAS6) | Q14393 | Cardiometabolic | 8.78 | 66.43 | ICC<80% |
| Hepatocyte growth factor (HGF) | P14210 | Inflammation | 1.93 | 90.72 |  |
| Hepatocyte growth factor receptor (MET) | P08581 | Cardiometabolic | 10.83 | 54.47 | ICC<80% |
| Ig lambda-2 chain C regions (IGLC2) | P0CG05 | Cardiometabolic | 3.83 | 79.26 | ICC<80% |
| Insulin-like growth factor-binding protein 3 (IGFBP3) | P17936 | Cardiometabolic | 5.74 | 85.43 |  |
| Insulin-like growth factor-binding protein 6 (IGFBP6) | P24592 | Cardiometabolic | 6.79 | 78.12 | ICC<80% |
| Integrin alpha-M (ITGAM) | P11215 | Cardiometabolic | 2.55 | 99.9 |  |
| Intercellular adhesion molecule 1 (ICAM1) | P05362 | Cardiometabolic | 4.13 | 82.32 |  |
| Intercellular adhesion molecule 3 (ICAM3) | P32942 | Cardiometabolic | 14.65 | 46.39 | ICC<80% |
| Interferon gamma (IFN-gamma) | P01579 | Inflammation |  |  | Low detection |
| Interleukin-1 alpha (IL-1 alpha) | P01583 | Inflammation |  |  | Low detection |
| Interleukin-10 (IL10) | P22301 | Inflammation | 4.94 | 97.61 |  |
| Interleukin-10 receptor subunit alpha (IL-10RA) | Q13651 | Inflammation | 2.99 | 99.8 |  |
| Interleukin-10 receptor subunit beta (IL-10RB) | Q08334 | Inflammation | 2.7 | 73.33 | ICC<80% |
| Interleukin-12 subunit beta (IL-12B) | P29460 | Inflammation | 2.62 | 95.93 |  |
| Interleukin-13 (IL-13)† | P35225 | Inflammation |  |  |  |
| Interleukin-15 receptor subunit alpha (IL-15RA) | Q13261 | Inflammation | 13.8 | 16.51 | ICC<80% |
| Interleukin-17A (IL-17A) | Q16552 | Inflammation | 20.09 | 89.65 |  |
| Interleukin-17C (IL-17C) | Q9P0M4 | Inflammation | 4.19 | 92.51 |  |
| Interleukin-18 (IL-18) | Q14116 | Inflammation | 2.09 | 95.53 |  |
| Interleukin-18 receptor 1 (IL-18R1) | Q13478 | Inflammation | 1.89 | 92.9 |  |
| Interleukin-2 (IL-2)‡ | P60568 | Inflammation |  |  | Low detection |
| Interleukin-2 receptor subunit beta (IL-2RB) | P14784 | Inflammation |  |  | Low detection |
| Interleukin-20 (IL-20) | Q9NYY1 | Inflammation |  |  | Low detection |
| Interleukin-20 receptor subunit alpha (IL-20RA) | Q9UHF4 | Inflammation | 5.88 | 97.79 |  |
| Interleukin-22 receptor subunit alpha-1 (IL-22 RA1) | Q8N6P7 | Inflammation |  |  | Low detection |
| Interleukin-24 (IL-24) | Q13007 | Inflammation | 1.39 | 99.76 |  |
| Interleukin-33 (IL-33) | O95760 | Inflammation |  |  | Low detection |
| Interleukin-4 (IL-4) | P05112 | Inflammation |  |  | Low detection |
| Interleukin-5 (IL5) | P05113 | Inflammation | 11.27 | 87.48 |  |
| Interleukin-6 (IL6) | P05231 | Inflammation | 5.94 | 98.04 |  |
| Interleukin-7 (IL-7) | P13232 | Inflammation | 4.35 | 99.12 |  |
| Interleukin-7 receptor subunit alpha (IL7R) | P16871 | Cardiometabolic | 19.8 | 72.54 | ICC<80% |
| Interleukin-8 (IL-8) | P10145 | Inflammation | 2.72 | 99.35 |  |
| Latency-associated peptide transforming growth factor beta-1 (LAP TGF-beta-1) | P01137 | Inflammation | 2.11 | 96.95 |  |
| Latent-transforming growth factor beta-binding protein 2 (LTBP2) | Q14767 | Cardiometabolic | 43.8 | 23.65 | ICC<80% |
| Leukemia inhibitory factor (LIF) | P15018 | Inflammation | 8.01 | 95 |  |
| Leukemia inhibitory factor receptor (LIF-R) | P42702 | Inflammation | 4.53 | 75.93 | ICC<80% |
| Leukocyte immunoglobulin-like receptor subfamily B member 1 (LILRB1) | Q8NHL6 | Cardiometabolic | 21.3 | 81.51 |  |
| Leukocyte immunoglobulin-like receptor subfamily B member 2 (LILRB2) | Q8N423 | Cardiometabolic | 9.42 | 77.47 | ICC<80% |
| Leukocyte immunoglobulin-like receptor subfamily B member 5 (LILRB5) | O75023 | Cardiometabolic | 7.68 | 76.02 | ICC<80% |
| Lithostathine-1-alpha (REG1A) | P05451 | Cardiometabolic | 3.85 | 90.18 |  |
| Liver carboxylesterase 1 (CES1) | P23141 | Cardiometabolic | 11.78 | 92.82 |  |
| Low affinity immunoglobulin gamma Fc region receptor II-a (FCGR2A) | P12318 | Cardiometabolic | 7.01 | 69.53 | ICC<80% |
| Low affinity immunoglobulin gamma Fc region receptor III-B (FCGR3B) | O75015 | Cardiometabolic | 5.91 | 89.83 |  |
| L-selectin (SELL) | P14151 | Cardiometabolic | 4.88 | 59.52 | ICC<80% |
| Lymphatic vessel endothelial hyaluronic acid receptor 1 (LYVE1) | Q9Y5Y7 | Cardiometabolic | 5.09 | 75.23 | ICC<80% |
| Lysosomal Pro-X carboxypeptidase (PRCP)† | P42785 | Cardiometabolic |  |  |  |
| Macrophage colony-stimulating factor 1 (CSF-1) | P09603 | Inflammation | 1.38 | 82.55 |  |
| Mannose-binding protein C (MBL2) | P11226 | Cardiometabolic | 3.2 | 95.31 |  |
| Mast/stem cell growth factor receptor Kit (KIT) | P10721 | Cardiometabolic | 8.29 | 74.83 | ICC<80% |
| Matrix metalloproteinase-1 (MMP-1) | P03956 | Inflammation | 1.3 | 99.02 |  |
| Matrix metalloproteinase-10 (MMP-10) | P09238 | Inflammation | 2.82 | 95.27 |  |
| Membrane cofactor protein (CD46) | P15529 | Cardiometabolic | 15.38 | 61.93 | ICC<80% |
| Membrane primary amine oxidase (AOC3) | Q16853 | Cardiometabolic | 7.19 | 78.53 | ICC<80% |
| Metalloproteinase inhibitor 1 (TIMP1) | P01033 | Cardiometabolic | 4.28 | 73.59 | ICC<80% |
| Microfibrillar-associated protein 5 (MFAP5)) | Q13361 | Cardiometabolic | 25.28 | 56.05 | ICC<80% |
| Monocyte chemotactic protein 1 (MCP-1) | P13500 | Inflammation | 1.86 | 88.52 |  |
| Monocyte chemotactic protein 2 (MCP-2) | P80075 | Inflammation | 1.98 | 97.96 |  |
| Monocyte chemotactic protein 3 (MCP-3) | P80098 | Inflammation | 6.04 | 93.71 |  |
| Monocyte chemotactic protein 4 (MCP-4) | Q99616 | Inflammation | 1.39 | 92.51 |  |
| Multiple epidermal growth factor-like domains protein 9 (MEGF9)) | Q9H1U4 | Cardiometabolic | 17.08 | 66.88 | ICC<80% |
| Natural killer cell receptor 2B4 (CD244) | Q9BZW8 | Inflammation | 2.53 | 91.48 |  |
| Neural cell adhesion molecule 1 (NCAM1)) | P13591 | Cardiometabolic | 8.48 | 67.23 | ICC<80% |
| Neural cell adhesion molecule L1-like protein (CHL1) | O00533 | Cardiometabolic | 7.88 | 72.29 | ICC<80% |
| Neurogenic locus notch homolog protein 1 (NOTCH1) | P46531 | Cardiometabolic | 10.29 | 59.44 | ICC<80% |
| Neuropilin-1 (NRP1) | O14786 | Cardiometabolic | 6.08 | 54.37 | ICC<80% |
| Neurotrophin-3 (NT-3) | P20783 | Inflammation | 10.54 | 70.54 | ICC<80% |
| Neurturin (NRTN) | Q99748 | Inflammation |  |  | Low detection |
| Neutrophil defensin 1 (DEFA1) | P59665 | Cardiometabolic | 3.3 | 99.67 |  |
| Neutrophil gelatinase-associated lipocalin (LCN2) | P80188 | Cardiometabolic | 23.23 | 44.35 | ICC<80% |
| Nidogen-1 (NID1) | P14543 | Cardiometabolic | 6.99 | 76.77 | ICC<80% |
| Oncostatin-M (OSM) | P13725 | Inflammation | 4.5 | 99.12 |  |
| Oncostatin-M-specific receptor subunit beta (OSMR) | Q99650 | Cardiometabolic | 6.33 | 67.29 | ICC<80% |
| Osteoprotegerin (OPG) | O00300 | Inflammation | 1.68 | 86.93 |  |
| Peptidyl-glycine alpha-amidating monooxygenase (PAM) | P19021 | Cardiometabolic | 11.13 | 63.05 | ICC<80% |
| Phospholipid transfer protein (PLTP) | P55058 | Cardiometabolic | 41.77 | 50.41 | ICC<80% |
| Plasma serine protease inhibitor (SERPINA5) | P05154 | Cardiometabolic | 2.81 | 88.45 |  |
| Platelet glycoprotein Ib alpha chain (GP1BA) | P07359 | Cardiometabolic | 11.62 | 76.01 | ICC<80% |
| Platelet-activating factor acetylhydrolase (PLA2G7) | Q13093 | Cardiometabolic | 15.01 | 62.61 | ICC<80% |
| Plexin-B2 (PLXNB2) | O15031 | Cardiometabolic | 13.86 | 60.99 | ICC<80% |
| Procollagen C-endopeptidase enhancer 1 (PCOLCE) | Q15113 | Cardiometabolic | 4.34 | 84.24 |  |
| Programmed cell death 1 ligand 1 (PD-L1) | Q9NZQ7 | Inflammation | 3.35 | 93.95 |  |
| Prolyl endopeptidase FAP (FAP) | Q12884 | Cardiometabolic | 138.24 | 40.43 | ICC<80% |
| Protein S100-A12 (EN-RAGE ) | P80511 | Inflammation | 6.8 | 97.68 |  |
| Receptor-type tyrosine-protein phosphatase S (PTPRS) | Q13332 | Cardiometabolic | 17.06 | 65.65 | ICC<80% |
| Regenerating islet-derived protein 3-alpha (REG3A) | Q06141 | Cardiometabolic |  |  | Low detection |
| Serum amyloid A-4 protein (SAA4) | P35542 | Cardiometabolic | 16.22 | 71.56 | ICC<80% |
| Signaling lymphocytic activation molecule (SLAMF1) | Q13291 | Inflammation | 7.54 | 86.07 |  |
| SIR2-like protein 2 (SIRT2) | Q8IXJ6 | Inflammation | 3.6 | 99.44 |  |
| SPARC-like protein 1 (SPARCL1) | Q14515 | Cardiometabolic | 14.22 | 61.17 | ICC<80% |
| STAM-binding protein (STAMPB) | O95630 | Inflammation | 2.7 | 99.5 |  |
| Stem cell factor (SCF) | P21583 | Inflammation | 1.1 | 96.6 |  |
| Sulfotransferase 1A1 (ST1A1) | P50225 | Inflammation | 3.41 | 99.64 |  |
| Superoxide dismutase [Cu-Zn] (SOD1) | P00441 | Cardiometabolic | 4.53 | 98.26 |  |
| T cell surface glycoprotein CD6 isoform (CD6) | Q8WWJ7 | Inflammation | 2.78 | 96.54 |  |
| T cell surface glycoprotein CD8 alpha chain (CD8A) | P01732 | Inflammation | 2.16 | 95.85 |  |
| T-cell immunoglobulin and mucin domain-containing protein 4 (TIMD4) | Q96H15 | Cardiometabolic | 10.88 | 67.71 | ICC<80% |
| T-cell surface glycoprotein CD5 (CD5) | P06127 | Inflammation | 2.45 | 95.56 |  |
| Tenascin (TNC) | P24821 | Cardiometabolic | 14.5 | 72.15 | ICC<80% |
| Tenascin-X (TNXB) | P22105 | Cardiometabolic | 7.32 | 70.45 | ICC<80% |
| Thrombospondin-4 (THBS4) | P35443 | Cardiometabolic | 4.89 | 90.32 |  |
| Thymic stromal lymphopoietin (TSLP) | Q969D9 | Inflammation |  |  | Low detection |
| Thyroxine-binding globulin (SERPINA7) | P05543 | Cardiometabolic | 5.42 | 81.94 |  |
| TNF-beta (TNFB) | P01374 | Inflammation | 2.82 | 92.65 |  |
| TNF-related activation-induced cytokine (TRANCE) | O14788 | Inflammation | 4.89 | 94.97 |  |
| TNF-related apoptosis-inducing ligand (TRAIL) | P50591 | Inflammation | 2.27 | 82.2 |  |
| Transcobalamin-2 (TCN2) | P20062 | Cardiometabolic | 9.44 | 74.44 | ICC<80% |
| Transforming growth factor alpha (TGF-alpha) | P01135 | Inflammation | 4.92 | 94.47 |  |
| Transforming growth factor beta receptor type 3 (TGFBR3) | Q03167 | Cardiometabolic | 20.31 | 89.85 |  |
| Transforming growth factor-beta-induced protein ig-h3 (TGFBI) | Q15582 | Cardiometabolic | 4.87 | 66.6 | ICC<80% |
| Trypsin-2 (PRSS2) | P07478 | Cardiometabolic | 9.35 | 91.82 |  |
| Tumor necrosis factor (Ligand) superfamily, member 12 (TWEAK) | O43508 | Inflammation | 2.01 | 90.13 |  |
| Tumor necrosis factor (TNF)* | P01375 | Inflammation | 5.4 |  |  |
| Tumor necrosis factor ligand superfamily member 14 (TNFSF14 ) | O43557 | Inflammation | 2.86 | 98.87 |  |
| Tumor necrosis factor receptor superfamily member 9 (TNFRSF9) | Q07011 | Inflammation | 2.74 | 87.9 |  |
| Tyrosine-protein kinase receptor Tie-1 (TIE1) | P35590 | Cardiometabolic | 7.83 | 69.33 | ICC<80% |
| Urokinase-type plasminogen activator (uPA) | P00749 | Inflammation | 1.81 | 86.93 |  |
| Uromodulin (UMOD) | P07911 | Cardiometabolic | 0.32 | 99.85 |  |
| Vascular cell adhesion protein 1 (VCAM1) | P19320 | Cardiometabolic | 6.72 | 66.96 | ICC<80% |
| Vascular endothelial growth factor A (VEGF-A) | P15692 | Inflammation | 1.84 | 95.38 |  |
| Vasorin (VASN) | Q6EMK4 | Cardiometabolic | 11.85 | 55.92 | ICC<80% |
| Vitamin K-dependent protein C (PROC) | P04070 | Cardiometabolic | 7.92 | 64.32 | ICC<80% |
